# Supplementary material for: A digital twin for parallel liquid-state nuclear magnetic resonance spectroscopy
Source: Commun Eng. 2024 Jun 29;3:90. doi: 10.1038/s44172-024-00233-0 (PMC11217505; doi:10.1038/s44172-024-00233-0)
Supplement: Supplementary file 2 — supplementary information [file 44172_2024_233_MOESM2_ESM.pdf]

Supplementary Information for

A digital twin for parallel liquid-state nuclear magnetic resonance spectroscopy

Mengjia He, Dilara Faderl, Neil MacKinnon*, Yen-Tse Cheng, Dominique Buyens, Mazin Jouda, Burkhard Luy, and Jan G. Korvink*

*Corresponding authors: neil.mackinnon@kit.edu, jan.korvink@kit.edu

Contents

Supplementary Note 1. Electromagnetic simulation flow 2

Supplementary Note 2. RF chain modeling 3

Supplementary Note 3. B_1 field simulation of the coil array 8

Supplementary Note 4. Sample-sample coupling via the radiation damping 11

Supplementary Note 5. Transfer fidelity of parallel pulse 12

Supplementary Note 6. Derivation for signal decomposition 14

Supplementary Note 7. Robustness of signal decomposition 16

Supplementary Note 8. Signal decomposition with SOBI method 18

Supplementary Note 9. Wall clock time of the optimal control 22

Supplementary Note 10. Spin dynamics simulation for multiple samples 23

Supplementary Note 11. Single channel optimal control 24

Supplementary Note 12. Variables index 27

Supplementary Note 13. Parallel NMR experiment setup 28

Supplementary Note 14. Chemical shifts of samples 30

Supplementary Note 1. Electromagnetic simulation flow

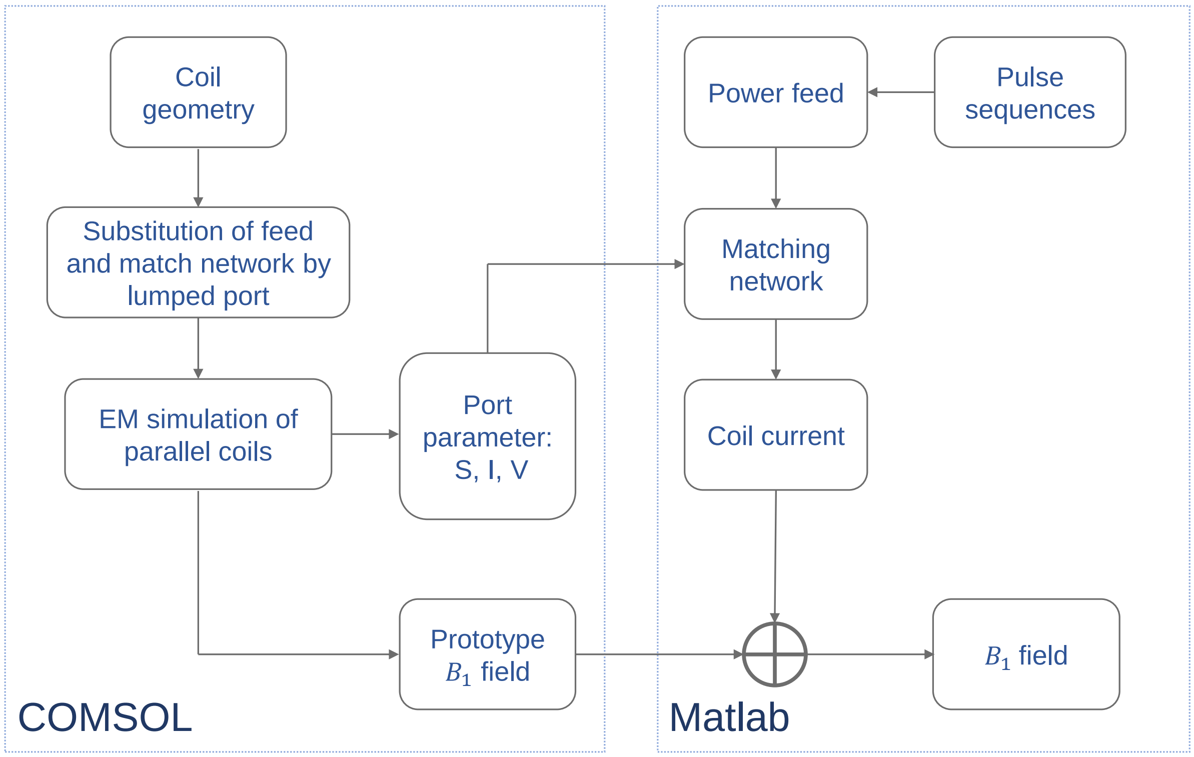

Figure S1 Electromagnetic simulation flow for a parallel radio frequency (RF) probe. For a specific frequency (or nucleus), the electromagnetic (EM) simulation is executed only once for the coil array, since the post-processing in Matlab can be executed repeatedly to obtain the radio frequency magnetic field (B_1) corresponding to different applied pulse sequences. S, I and V are the S-parameters, currents and voltages, respectively.

Supplementary Note 2. RF chain modeling

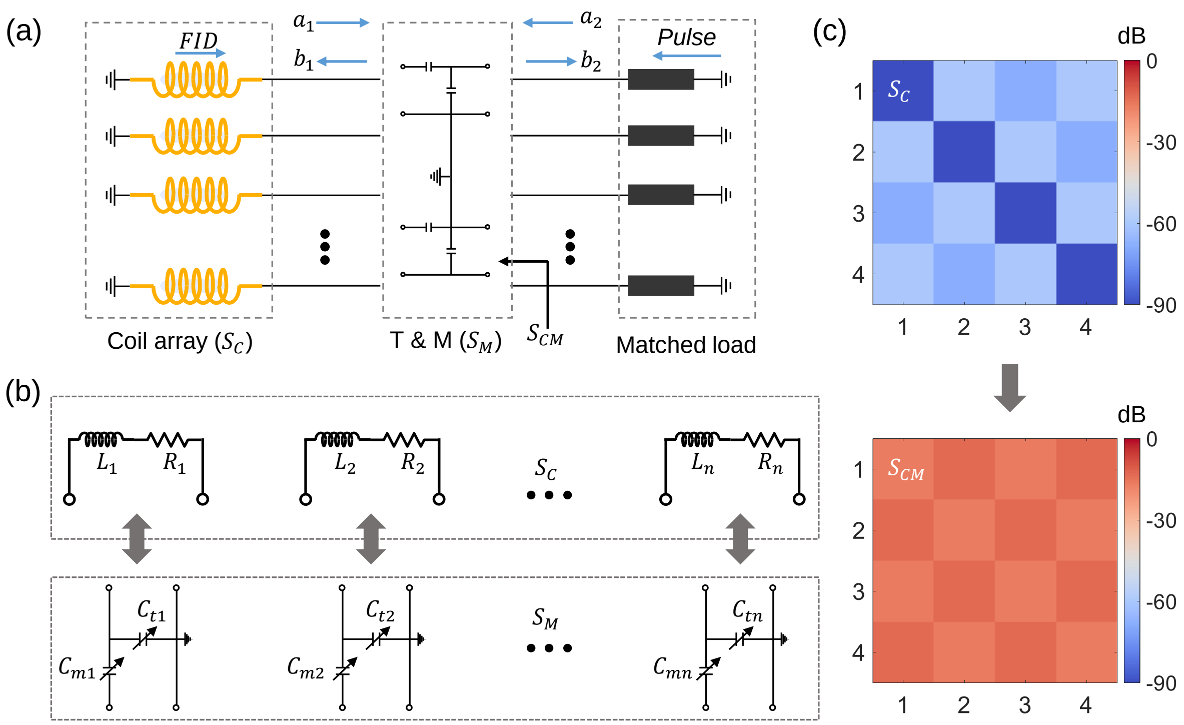

Figure S2 Combination of coil array and tuning and matching (T&M) network using S-parameters. (a) Block diagram of the parallel probe including a mutually coupled coil array, matching network, and matched loads. The coils could have different configurations. The free-induction-decay is denoted FID, the S_C, S_M, and S_{CM} are the S-matrices of the coil array, the T&M network, and their combination, respectively. The forward waves are denoted a_1, a_2, and the reverse waves b_1, b_2. (b) A schematic diagram shows the port connection between the coil and the T&M network. A serial connection of L and R forms the circuit model of the coil, and C_t and C_m denote the tuning and matching capacitances, respectively. (c) Amplitude of the S-matrix for a 4-solenoid array: coil array (top, the diagonal elements are ignored for comparison) and coil array combined with T&M network (bottom), the off-diagonal elements display an increase of more than 40 dB.

As shown in Fig. S1, the RF chain model was built in Matlab to calculate the B_1 field. Fig. S2(a) shows the port connection between the coil array and the tuning and matching (TM) network and the signals' pathway. In the RF chain modeling, the first step is designing the tuning and matching network. Considering that coil coupling is sufficiently weak, here the coupling in the solenoid coil array is -60 dB ($S_{C,21}$); the typical coupling for the 2-saddle coil is below -70 dB [1]; and coupling between striplines shall be smaller. So each coil is individually

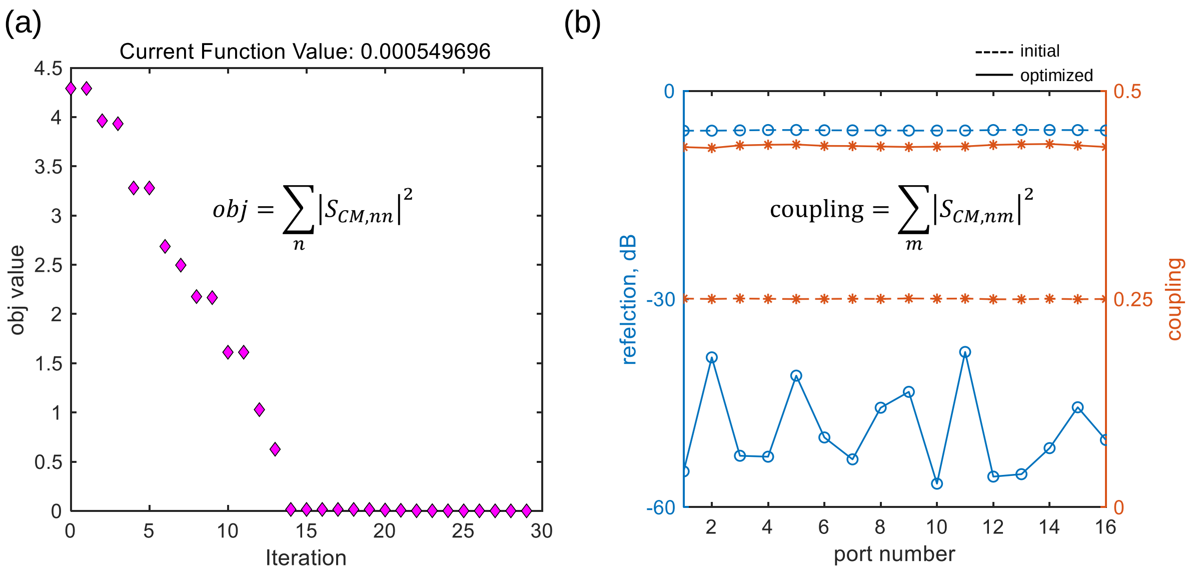

Figure S3 Optimization of the tuning and matching network for a 16-solenoid array, to minimize the total reflection coefficient, the diagonal element of S_{CM}. (a) The convergence of the objective function, given by the Matlab `fminsearch` function, 'obj' is the objective function. (b) The reflection and total coupling ratio at each port. Dashed lines denote the initial values produced by individual tuning and matching, and solid lines denote the optimized values.

tuned and matched using the diagonal elements of S_C, generating the initial TM network. When the coupling increases and causes an undesirable mismatch, such as $S_{CM,11} > -20$ dB as shown in Fig. S4(a), we adjusted the tuning and matching capacitances to fix it. To optimize the capacitances with the target of minimizing the reflection, we use the Nelder-Mead simplex algorithm[2], which is a derivative-free method used for unconstrained optimization problems, and the algorithm is already implemented in Matlab (`fminsearch`). Fig. S3 displays the optimized results for a 16 solenoid array, the reflection at each port is below -30 dB. However, the coupling ratio has greatly enhanced, this issue shall be addressed by the pulse compensation. Note that applying optimization needs the coil array S matrix, one may need to measure this S matrix in the experiments.

Next, we combine the coil array and TM network using S parameters. Representing the optimized matching network using a block matrix S-parameter description

$$S_M = \begin{bmatrix} S_{11} & S_{12} \\ S_{21} & S_{22} \end{bmatrix}, \tag{S1}$$

where 1 and 2 refer to connecting ports and free ports, respectively. According to the following

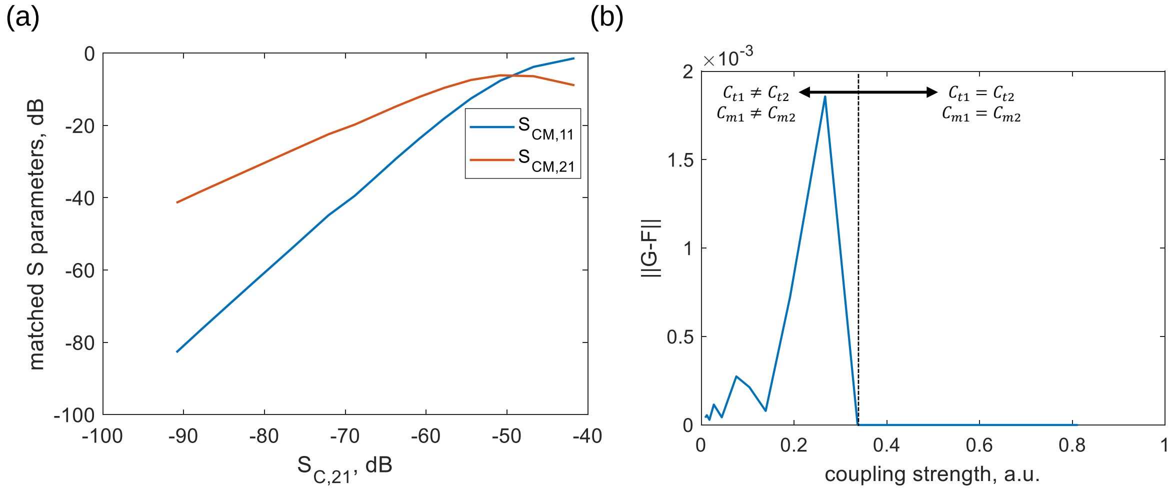

Figure S4 Coupling ratio sweep of a 2 solenoid array. (a) Matched S-parameters as a function of $S_{C,21}$ in decibels. (b) The difference between the normalized G and F matrices, i.e., the Frobenius norm of $G_{\text{norm}} - F_{\text{norm}}$ as a function of matched coupling strength in arbitrary units. The dashed line separates the two choices for tuning and matching, i.e., nonidentical and identical capacitances.

equations

$$\begin{aligned} b_1 &= S_{11}a_1 + S_{12}a_2, \\ b_2 &= S_{21}a_1 + S_{22}a_2, \\ a_1 &= S_C b_1, \end{aligned} \tag{S2}$$

the combined matrix at the matching network terminal was derived as[3]

$$S_{CM} = S_{22} + S_{21}(E - S_C S_{11})^{-1} \cdot S_C S_{12}, \tag{S3}$$

At the excitation stage, the incident and reflection wave at coil terminals were given by

$$\begin{aligned} a_1 &= (E - S_C S_{11})^{-1} \cdot S_C S_{12} \cdot a_2, \\ b_1 &= S_C^{-1}(E - S_C S_{11})^{-1} \cdot S_C S_{12} \cdot a_2. \end{aligned} \tag{S4}$$

Note that a_1 and b_1 are defined at the TM network port, so concerning the coil port, a_1 corresponds to the reverse wave and b_1 corresponds to the forward wave. The current and voltage on the coils are calculated as

$$\begin{aligned} I_C &= \frac{1}{\sqrt{Z_0}}(b_1 - a_1), \\ V_C &= \sqrt{Z_0}(b_1 + a_1). \end{aligned} \tag{S5}$$

The RF pulse p is regarded as the excitation signal a_2, we conclude that the transform matrix from a_2 to coil current I_C is given by

$$F = \frac{1}{\sqrt{Z_0}}(S_C^{-1} - E) \cdot (E - S_C S_{11})^{-1} \cdot S_C S_{12}. \tag{S6}$$

At the reception stage, the output port of the matching network is connected to low-noise amplifiers whose input can be regarded as a matched load. The FID is defined as an open-circuit voltage V_{1on} at the coil terminal. Regards the coil array, in the open-circuit condition, we have

$$\begin{aligned} a_1 &= b_1, \\ V_{1on} &= \sqrt{Z_0}(a_1 + b_1). \end{aligned} \tag{S7}$$

and in the meanwhile, the termination equation can also be given in a general load case,

$$a_1 = b_R + S_c b_1 \tag{S8}$$

where b_R is the FID-induced reverse wave. So we represent b_R with the open-circuit voltage V_{1on},

$$b_R = \frac{1}{2\sqrt{Z_0}}(E - S_C)V_{1on} \tag{S9}$$

Next, when the TM network is terminated with matched load, the voltage gain G, which is defined as $G = V_2/V_{1on}$. is calculated by combining Eq. (S2-S3, S7-S9)

$$G = \frac{1}{2} S_{21} \cdot (E - S_C \cdot S_{11})^{-1} \cdot (E - S_C). \tag{S10}$$

The matrix F quantifies the excitation coupling, and G quantifies the reception coupling. When each coil is individually tuned and matched, and the pure coil coupling is very weak, i.e., the off-diagonal elements of S_C are close to zero, the two matrices only differ by a constant number, i.e., $G \approx \sqrt{Z_0}/2 \cdot F$, as shown in Fig. S4(b). If all the coils have the same geometry and close impedance in a practical case, they can be tunned and matched with the same capacitance sets, as we did in the optimization for a strong coupling case, the S_{11} and S_{12} are exactly proportional to the identity matrix, then $F_{\text{norm}} = G_{\text{norm}}$.

Note that noise coupling flows the same way as FID coupling before the signals transfer into the LNA. Here we replaced the LNA with the matched loads and suppose the noise is contributed by the coil array. The covariance of the noise voltage

$$\hat{R}_n = 4kTB \cdot Re[Z_C] \tag{S11}$$

where k is Boltzmann's constant, T is the coil temperature in kelvins, Z_C is the coil array impedance matrix, and B is receiving bandwidth depending on the spectrometer. So the noise covariance on the load is

$$\hat{V}_n = G^* \hat{R}_n G^T \tag{S12}$$

The SNR on channel m is given by

$$SNR_m = \frac{\hat{V}_{s,mm}}{\hat{V}_{n,mm}} \tag{S13}$$

where the signal covariance is $\hat{V}_s = G^* [V_{1on}^* V_{1on}^T] G^T$.

Supplementary Note 3. B_1 field simulation of the coil array

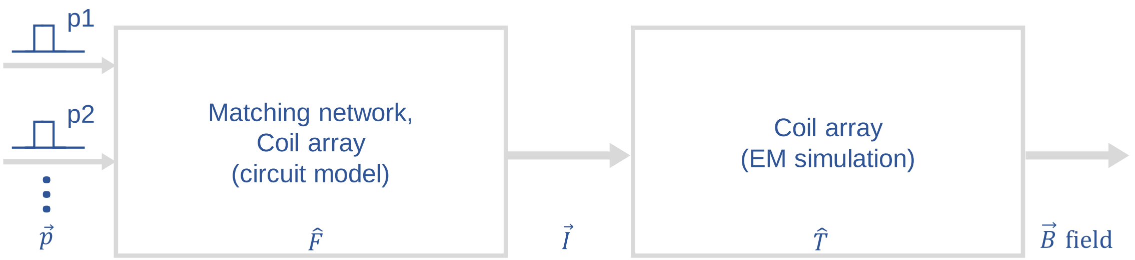

Figure S5 Flow of the parallel B_1 field calculation, the p vector indicates the pulse, I is the coil current, F and T are matrices representing the excitation gain at the circuit level and field level, respectively.

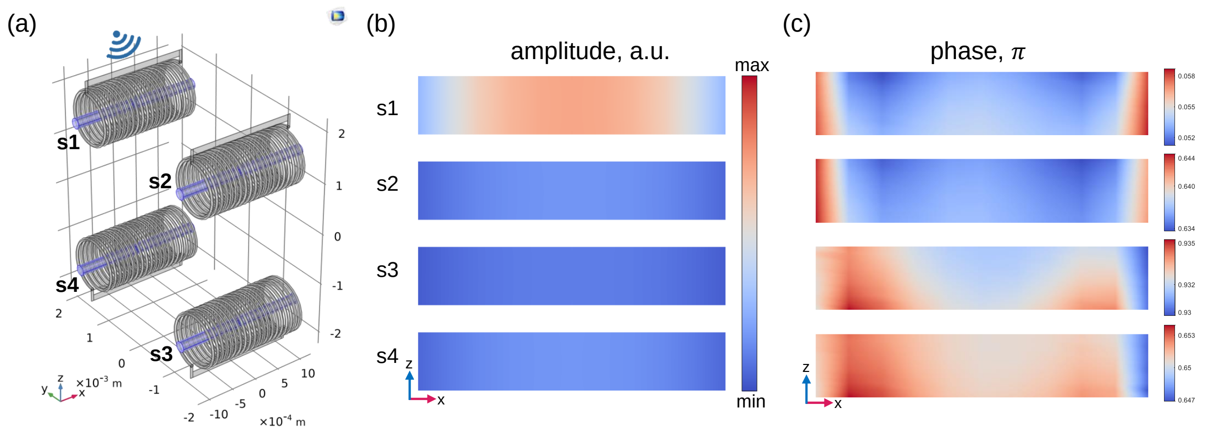

Figure S6 B_1 field of a 4 solenoid array. (a) The geometry of the solenoid array. Blue: water sample, substituted by a cylinder with radius $r_s = 0.1$ mm, length $l_s = 2.5$ mm. Gray: solenoid coil array, with radius $r_c = 0.5$ mm, length $l_c = 2.2$ mm with 20 turns, the radius of the wire is 0.03 mm, the coil distances in y and z direction are both 3 mm. (b-c) Amplitude (b) and phase (c) of the transmit B_1 field ($B_t = B_x + iB_y$) in the cross-plane (xoz) inside the sample. Only the first channel is excited from the tuning and matching network terminal, i.e., $p = (1,0,0,0)^T$.

Fig. S5 shows how the B_1 field is calculated. Under parallel pulse excitation, the coils' temperature shift was ignored, so the impedance match condition is preserved and the coil array is a linear and time-invariant system. We use the RF chain model built above to calculate the coil currents. According to the superposition principle, the B_1 field is the sum of each sub-component B_1. This sub B_1 is produced by an individual coil and proportional to the coil

current. The position-dependent B_1 is calculated from the follow equation

$$\boldsymbol{B_1} = T \cdot \begin{pmatrix} I_1 \\ I_2 \\ \vdots \\ I_n \end{pmatrix} = T \cdot F \cdot \begin{pmatrix} p_1 \\ p_2 \\ \vdots \\ p_n \end{pmatrix}, \tag{S14}$$

where the row dimension of T is non-determined, depending on the number of voxels considered. If n voxels are selected from n samples when calculating the T matrix, then T becomes an $n*n$ matrix. The F is an $n*n$ matrix, and can be calculated from Eq. S6. The T matrix can be calculated in the following way: through an EM simulation in COMSOL, the B field is calculated together with n port currents I, and n port sweeps gives n different $(B-I)$ combinations. For a specific position, we have

$$\begin{pmatrix} I_{11} & I_{12} & \dots & I_{1n} \\ I_{21} & I_{22} & \dots & I_{2n} \\ \vdots & \vdots & \ddots & \vdots \\ I_{n1} & I_{n2} & \dots & I_{nn} \end{pmatrix} \cdot T = \begin{pmatrix} B_1 \\ B_2 \\ \vdots \\ B_n \end{pmatrix}. \tag{S15}$$

The linear coefficients in the T vector are calculated from $T = I^{-1} \cdot B$.

Fig. S6 gives the B_1 field pattern of a 4-solenoid array, when only the first channel is excited, i.e., $p = (1,0,0,0)^T$. The amplitude of the simulated F and T matrix are given as following

$$\begin{aligned} |F| &= \begin{pmatrix} 0.4266 & 0.1084 & 0.0743 & 0.1089 \\ 0.1084 & 0.4265 & 0.1087 & 0.0743 \\ 0.0743 & 0.1088 & 0.4265 & 0.1088 \\ 0.1090 & 0.0744 & 0.1089 & 0.4266 \end{pmatrix}, \\ |T| &= 10^{-5} \cdot \begin{pmatrix} 1033 & 5.9902 & 2.0330 & 5.4047 \\ 5.9893 & 1033 & 5.4036 & 2.0342 \\ 2.0520 & 5.3936 & 1034 & 5.9281 \\ 5.3969 & 2.0536 & 5.9265 & 1033 \end{pmatrix}. \end{aligned} \tag{S16}$$

Here, each row of the T matrix is averaged over one sample consisting of 72 voxels. Since the ratio between off-diagonal and diagonal elements represents the coupling strength, we can see that the circuit-level coupling strength (F) is of order 10^{-1}, and the field spillover (T) is of order 10^{-3}, much smaller than the former.

To precisely model the coil current and B_1 field in the time domain, one may calculate the transient effect using the impulse response function[4] under linear time-invariant (LTI) system approximation. In routing NMR hardware, the transmission channel's bandwidth significantly exceeds the pulse bandwidth, resulting in insignificant transient effects. Therefore, it is customary to omit these transient effects during pulse design.

Supplementary Note 4. Sample-sample coupling via the radiation damping

The radiation damping is usually interpreted as that the FID signals on the coil generate an additional RF field B_{rad}, which in turn causes the magnetization to rotate toward the equilibrium state[5]. Considering that FID signals can transfer between channels, so the FID induced by one sample can excite an B_{rad} field in other samples, here we quantify this sample-sample coupling.

We assume that radiation-damping field strength can be exactly determined by the FID current on the coil, so the sample-sample coupling ratio via radiation damping is quantified by the current. Supposing that FID current on the primary coil and coupled coil as I_1 and I_2, respectively. The I_2/I_1 can be calculated through RF chain modeling in S2, i.e., supposing an initial open-circuit voltage $V_{1on} = [1,0]^T$, and calculating the coil current vector I_C, considering that the TM network is terminated with matched loads. If the primary radiation damping field is 50 Hz, the coupled field B_{rad} is given by

$$B_{\text{rad}} = 50 \text{ Hz} \cdot \frac{I_2}{I_1}, \tag{S17}$$

and shown in the following,

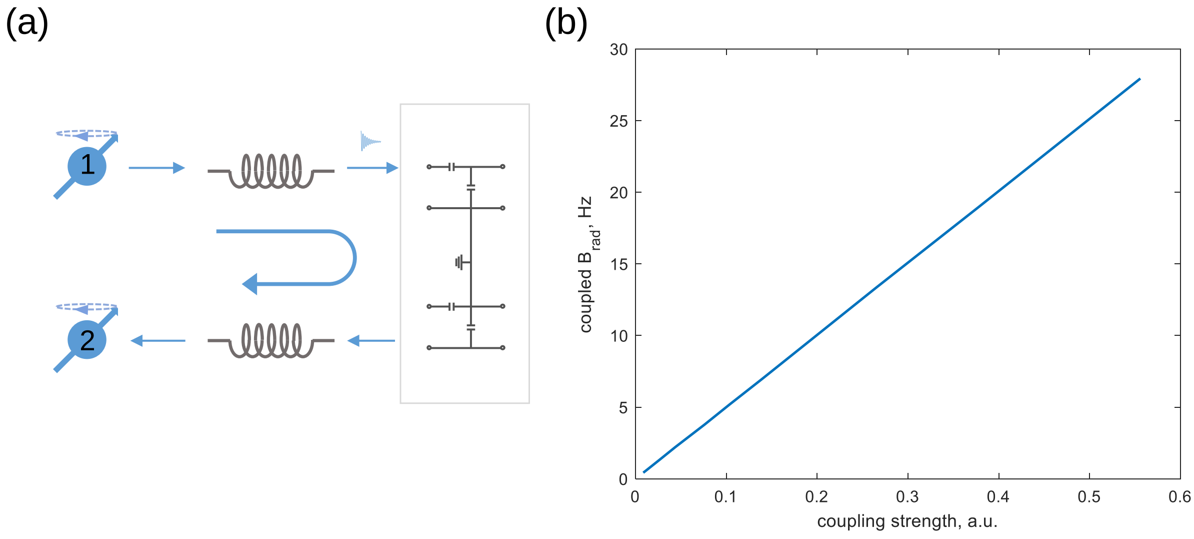

Figure S7 Quantification of the sample-sample coupling via the radiation damping effect.

Supplementary Note 5. Transfer fidelity of parallel pulse

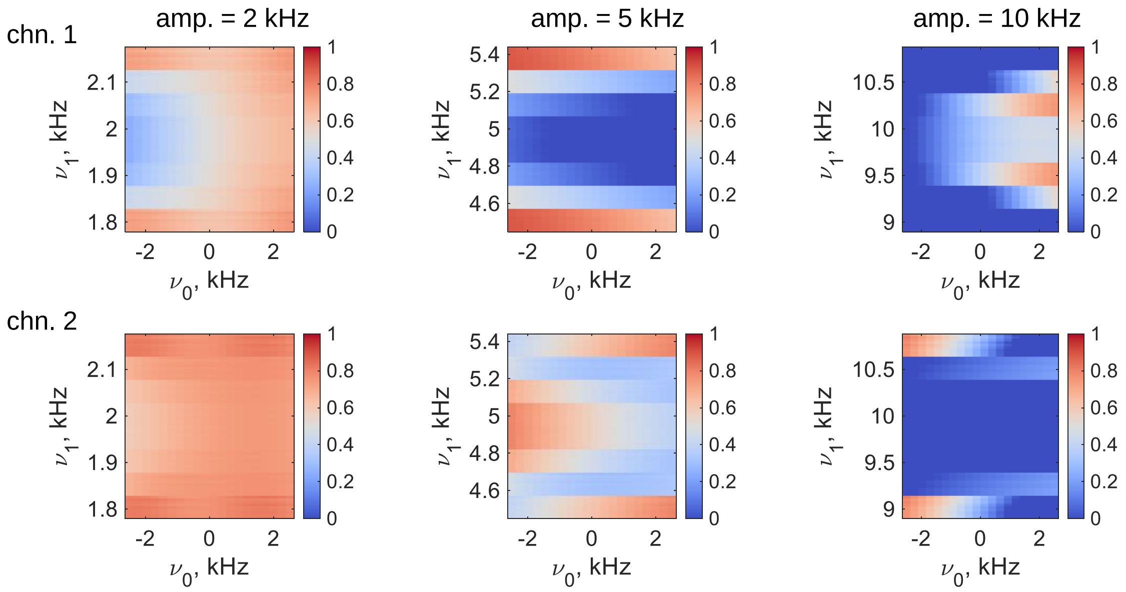

Figure S8 The transfer fidelity of the optimal control pulse applied in parallel was assessed across a range of pulse power levels, with a fixed coupling strength of c.s. = 0.24. The pulse duration is 2 ms, divided into 100 time steps. The fidelity diagram gives the transfer efficiency of a 2-channel cooperative pulse, each channel transfers one proton spin from I_z to I_x, taking into account the resonance offset and B_1 inhomogeneity. Two channels were indicated as chn. 1 and chn. 2 respectively. The ν_0 and ν_1 represent the resonance offset and radio frequency amplitude respectively, the amp. represents the nominal amplitude.

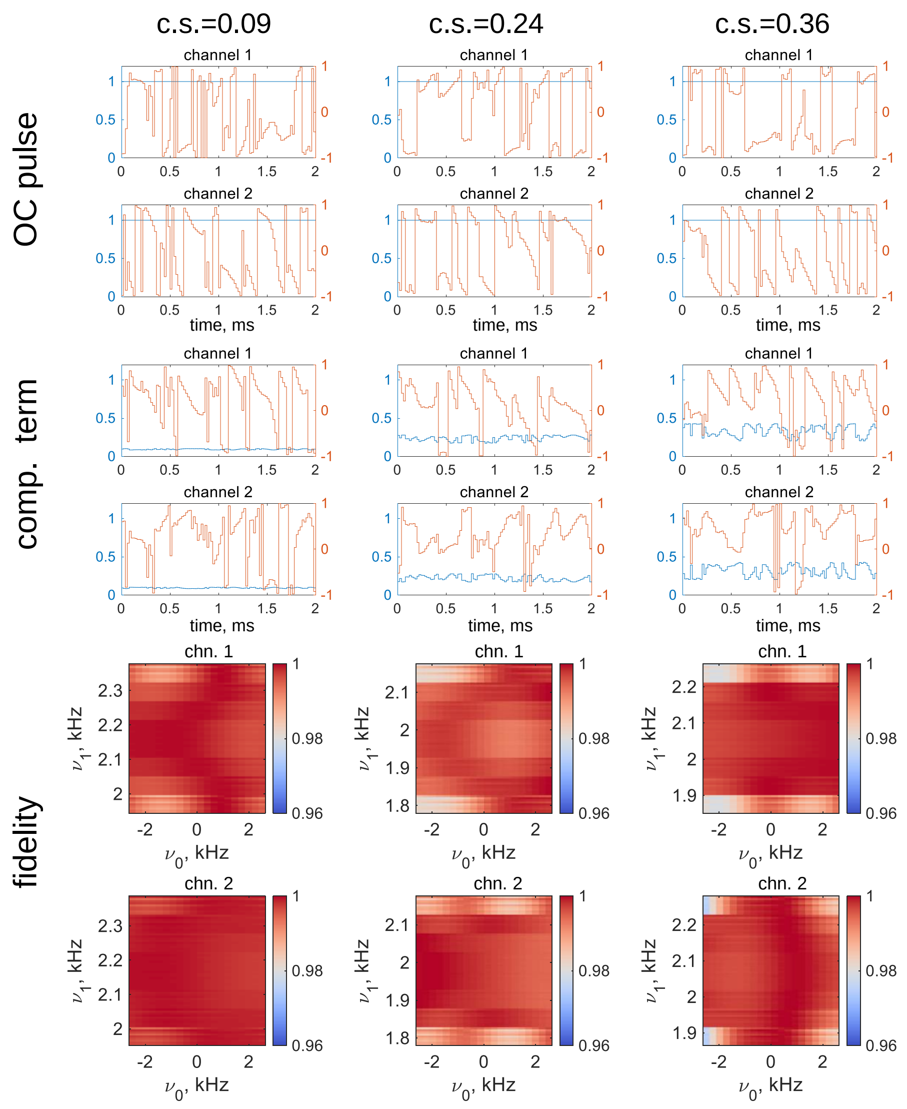

Figure S9 The waveform of the optimal control pulse and compensation term, and the transfer fidelity of the cooperative pulse across a range of radio frequency coupling strengths (c.s. $= |F_{12}/F_{11}|$). In the waveform plots, the blue lines represent amplitude while the orange lines represent phase (π). The spin system and pulse duration were the same as in Fig. S8. Two channels were indicated as chn. 1 and chn. 2 respectively. The ν_0 and ν_1 represent the resonance offset and radio frequency amplitude respectively. Optimal control is denoted by OC, and comp. denotes compensation. Frequencies are specified in kHz.

Supplementary Note 6. Derivation for signal decomposition

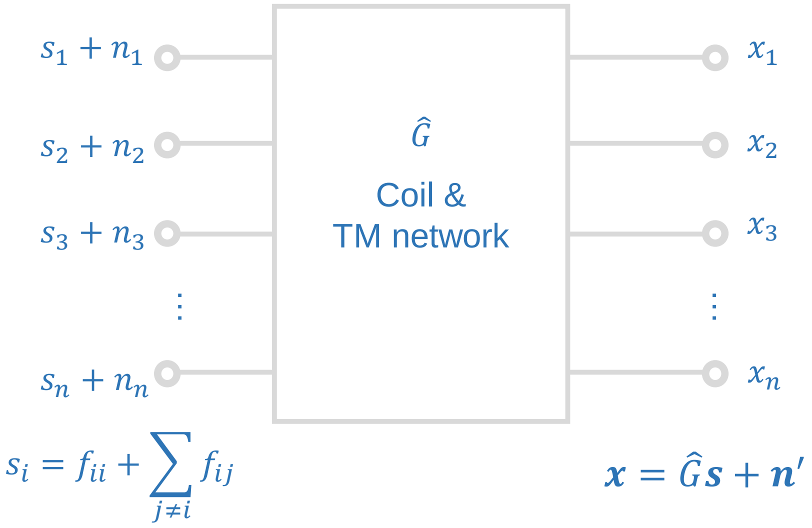

Figure S10 View for signal transmission in the reception stage. Here TM denotes tuning and matching, f_{ij} is the free-induction-decay (FID) on channel i from sample j, s_i is total FID on channel i, n is the noise, G is the reception gain matrix, x is the detected signal, and n' is the modified noise.

Fig. S10 gives an overview of signal composition in the reception stage, here we provide a rigorous derivation. The gain of the combined coil and matching network has been concluded in Eq. S10, which relates the received signal at the TM network output to the coils' open-circuit voltage induced by the FID. For convenience, we ignore the noise term and pick it up in the end. We write the received signal as an individual contribution plus a coupled contribution

$$\begin{pmatrix} x_1 \\ x_2 \\ \vdots \\ x_n \end{pmatrix} = G \cdot \begin{pmatrix} f_{11} \\ f_{22} \\ \vdots \\ f_{nn} \end{pmatrix} + G \cdot \begin{pmatrix} \sum_{j \neq 1} f_{1j} \\ \sum_{j \neq 2} f_{2j} \\ \vdots \\ \sum_{j \neq n} f_{nj} \end{pmatrix}, \tag{S18}$$

where the signal in the first channel can be further written as

$$x_1 = G_{11}(f_{11} + \sum_{j \neq 1} f_{1j}) + G_{12}(f_{22} + \sum_{j \neq 2} f_{2j}) + \ldots + G_{1n}(f_{nn} + \sum_{j \neq n} f_{nj}). \tag{S19}$$

Neglecting the second-order small terms in Eq. S19:

$$\begin{aligned} x_1 &\approx G_{11}f_{11} + (G_{11}f_{12} + G_{12}f_{22}) + ... + (G_{11}f_{1n} + G_{1n}f_{nn}) \\ &= G_{11}f_{11} + (G_{12} + R_{12}G_{11})f_{22} + (G_{13} + R_{13}G_{11})f_{33} + ... + (G_{1n} + R_{1n}G_{11})f_{nn}, \end{aligned} \tag{S20}$$

where

$$\begin{aligned} R_{ij} &= \frac{f_{ij}}{f_{jj}} = \frac{\int_{sample-j} B_{ij}\, dV_j}{\int_{sample-j} B_{jj}\, dV_j}, \\ f_{ij} &= \int_{sample-j} i\omega_0 M_j \cdot B_{ij}\, dV_j. \end{aligned} \tag{S21}$$

Note that Eq. S21 supposes a uniform magnetization, for example, $M_j = M_{\mathrm{x}}$ for an individual sample at the acquisition stage, R_{ij} indicates the ratio of the receiving B_1 field between coil i and coil j while detecting the j-th sample, and the reception phase from the sample to the coil was included in the receiving B_1 field. Following the approximation in Eq. S20, we obtain the simplified signal equation

$$\begin{aligned} G_{11}f_{11} + (G_{12} + R_{12}G_{11})f_{22} + (G_{13} + R_{13}G_{11})f_{33} + ... + (G_{1n} + R_{1n}G_{11})f_{nn} &= x_1, \\ (G_{21} + R_{21}G_{22})f_{11} + G_{22}f_{22} + (G_{23} + R_{23}G_{22})f_{33} + ... + (G_{2n} + R_{2n}G_{22})f_{nn} &= x_2, \\ &..., \\ (G_{n1} + R_{n1}G_{nn})f_{11} + (G_{n2} + R_{n2}G_{nn})f_{22} + ... + G_{nn}f_{nn} &= x_n. \end{aligned} \tag{S22}$$

Marking $G'_{ii} = G_{ii}$, $G'_{ij} = G_{ij} + G_{ii}R_{ij}$ and picking up the noise gives

$$\boldsymbol{x} = G' \cdot \boldsymbol{f} + \boldsymbol{n}', \tag{S23}$$

where $\boldsymbol{f} = (f_{11}, f_{22}, ..., f_{nn})^T$, so the primary FIDs f_{ii} can be extracted by solving Eq. S23.

In practice, We employ the blind source separation (BSS) method[6] to recover the uncoupled signals. In the BSS model, $\boldsymbol{f}$ is the source signals, $\boldsymbol{x}$ is the detected signals and $\boldsymbol{n}'$ is the noise. Note that in Eq. S23, we substitute $\boldsymbol{s}$ with $\boldsymbol{f}$ by neglecting the second-order small terms. Specifically, certain cross-coupling terms f_{ij}, are omitted. In this sense, the practical source signals are mutually correlated, and the correlation level depends on the sensitivity ratio R_{ij}, which is of order 10^{-3} (T matrix) for the solenoid coil array provided in supplementary S3. Its presence can introduce separation errors when utilizing the BSS method.

Supplementary Note 7. Robustness of signal decomposition

B_1 **field shift.** Since the B_1 field contributes to the signal decomposition but cannot be exactly predicted, here we study the decomposition efficiency under the B_1 field shift. Regarding this task, we calculate the FIDs with correctly predicted B_1 and deliberately shift their value when doing signal decomposition. The shifted B_1 is given by

$$B_1^{'} = B_1 \cdot (1 + \alpha \cdot \chi), \tag{S24}$$

where χ is a random number obeying a normal Gaussian distribution, i.e., $\chi \sim N(0,1)$, and α is the shift amplitude. Fig. S11 gives the signal decomposition results for two channels when shifting the B_1 field. When $\alpha > 100$, corresponding to a completely wrong prediction, the interference cannot be thoroughly removed from the signal, which indicates that the field-level combination makes a minimal contribution to signal decomposition.

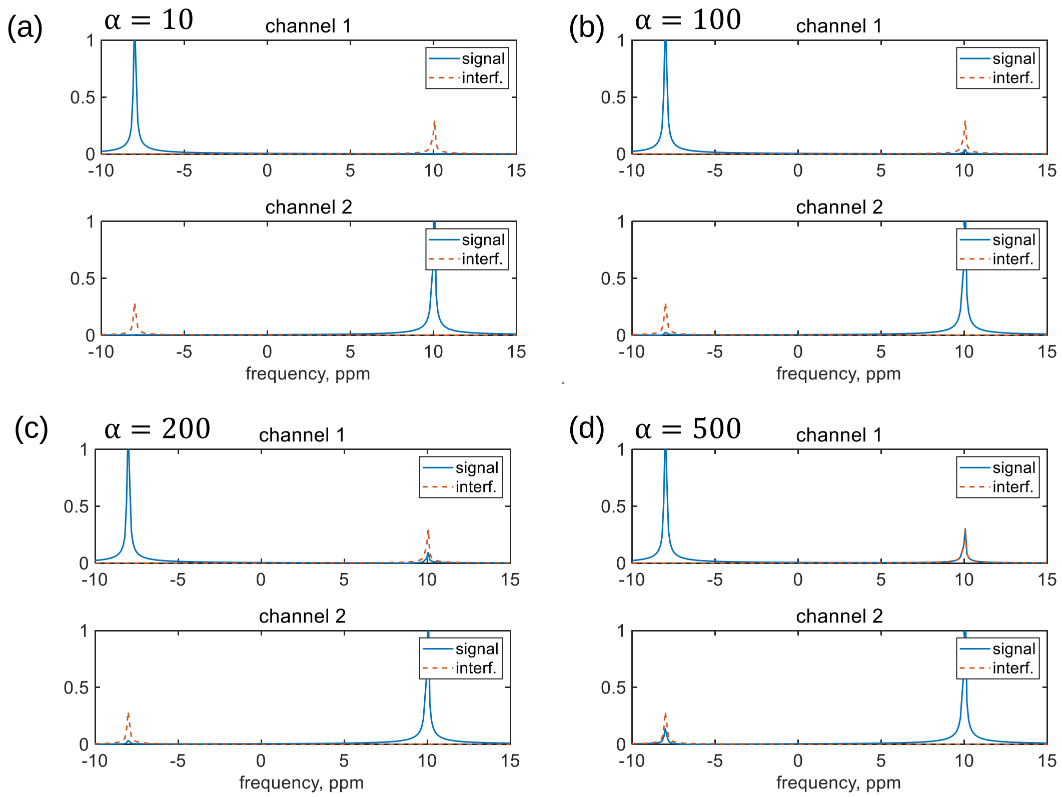

Figure S11 The 2-channel signal decomposition resulting when shifting the B_1 field with different amplitudes. The primary signal ('signal') and interfering coupled signal ('interf.') were plotted together. The shift amplitude is denoted by α, and ppm $\equiv$ parts-per-million.

Network gain shift. In the same way, we study the decomposition efficiency under the network gain G shift. We calculate the FIDs with correctly predicted gain and deliberately shift G when doing signal decomposition. Each element in G is given by

$$G'_{ij} = G_{ij} \cdot (1 + \alpha \cdot \chi), \tag{S25}$$

where χ is a random number obeying a normal Gaussian distribution, i.e., $\chi \sim N(0,1)$, and α is a shift amplitude. Fig. S12 gives the signal decomposition results for two channels when shifting the gain matrix. When $\alpha = 0.05$, no clear interference peaks exist on the signals. If α increases to 0.1, the interference peaks become remarkable. The decomposition is sensitive to the gain matrix because circuit-level combination contributes mostly to signal composition.

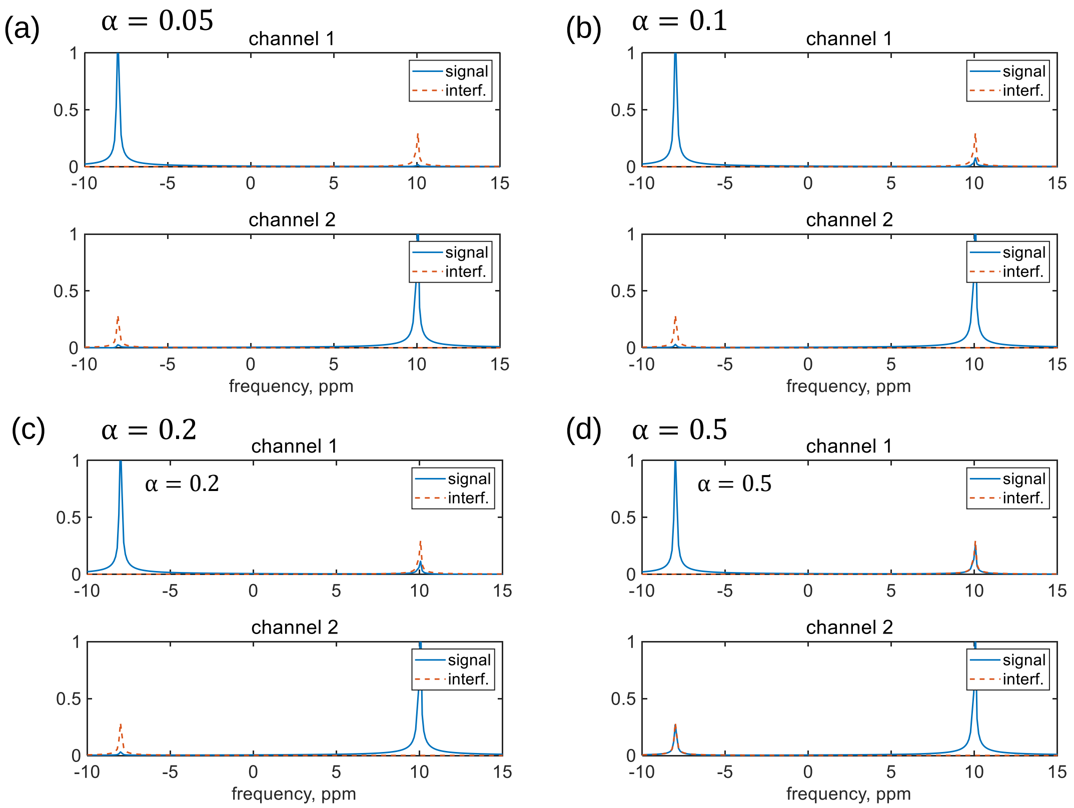

Figure S12 The 2-channel signal decomposition resulting when shifting the gain matrix G by different amplitudes. The primary signal ('signal') and the interfering coupled signal ('interf.') were plotted together. The shift amplitude is denoted by α, and ppm $\equiv$ parts-per-million.

Supplementary Note 8. Signal decomposition with SOBI method

Test using experimental data.

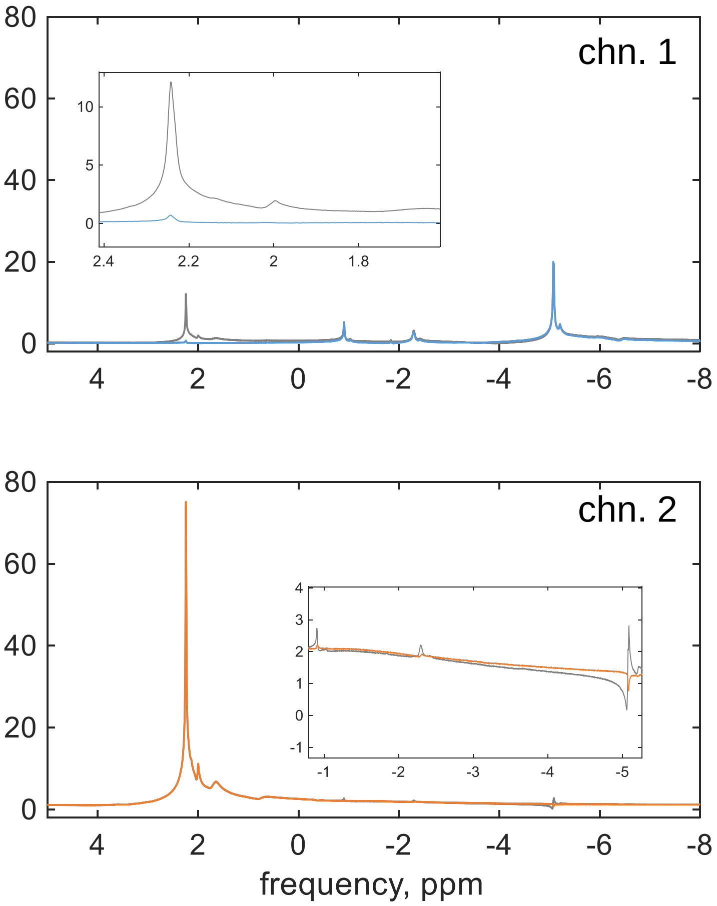

Figure S13 Signal decomposition of experimental 2-channel signals. Acetone is the sample in channel 1, while isopropanol is in channel 2. A global shim, set to 1000 Hz/cm along the x-axis, was applied to separate the two channels. A total of 256 scans were used. Two channels were indicated as chn. 1 and chn. 2, respectively. Here ppm $\equiv$ parts-per-million.

Spectral resolution. The signal decomposition faces difficulties with those signals with low SNR and small spectral differences (Δf). To study the resolution, each source shall contain only one frequency component. Because multiple components can introduce additional information for identifying coupling, potentially surpassing the resolution limit, as illustrated in Figure S15. Figure S14 gives a signal decomposition of an 8-channel system using simulated FID assuming homogeneous B_0, with the minimal $\Delta f = 3$ Hz and local SNR = 30 dB. Table 1 gives the resolution as the SNR increases. A higher SNR results in an enhanced resolution.

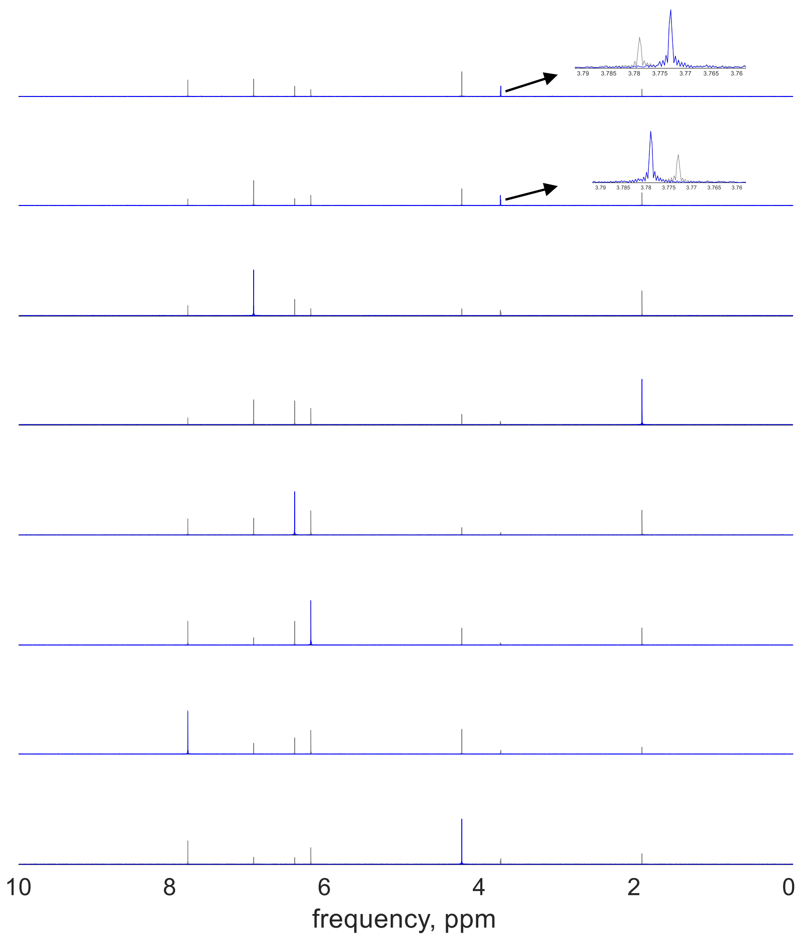

Figure S14 Signal decomposition of an 8-channel system using simulated data. Each channel is assigned with one spin, i.e., one chemical shift. The original signal is represented in grey, the split signal is represented in blue. Two signals with a minimal $\Delta f = 0.006$ ppm (3 Hz) and local signal-to-noise ratio SNR = 30 dB are separated, as illustrated in the zoomed-in view at the top. Here ppm $\equiv$ parts-per-million.

SNR, dB	20	23	25	30	35	40	45
resolution, Hz	25	17.5	3.5	3.0	2.5	1.5	1.0

Supplementary Table 1 Spectral resolution as the signal-to-noise (SNR) increases, assuming homogeneous magnetic field B0.

Signal decomposition for 8-channel with complex signals and averaged SNR=30 dB is given in Figure S15, the following table lists the corresponding chemical shifts. Note that two signals in channel-2 and channel-4 are very close (8.82 and 8.821 ppm), the SOBI method estimates the demixing matrix considering the overall frequency character, so it doesn't matter while a

few signals have tiny Δf.

channel	chemical shift, ppm
1	7.01, 2.97
2	0.87, 5.99, 2.54, 6.84, 2.22, 0.92, 8.821
3	8.98, 1.88, 6.56, 5.96, 8.87
4	5.04, 3.21, 5.47, 7.25, 5.74, 6.65, 8.820, 8.74
5	0.67, 8.56, 5.26, 2.53, 5.80, 2.30, 8.41
6	9.45, 5.54, 9.28, 6.20, 1.74, 5.10
7	0.86, 8.08, 1.36
8	5.70, 8.34, 3.28, 7.82, 2.62

Supplementary Table 2 Randomly generated chemical shifts for 8 channels in Figure S15, where ppm $\equiv$ parts-per-million.

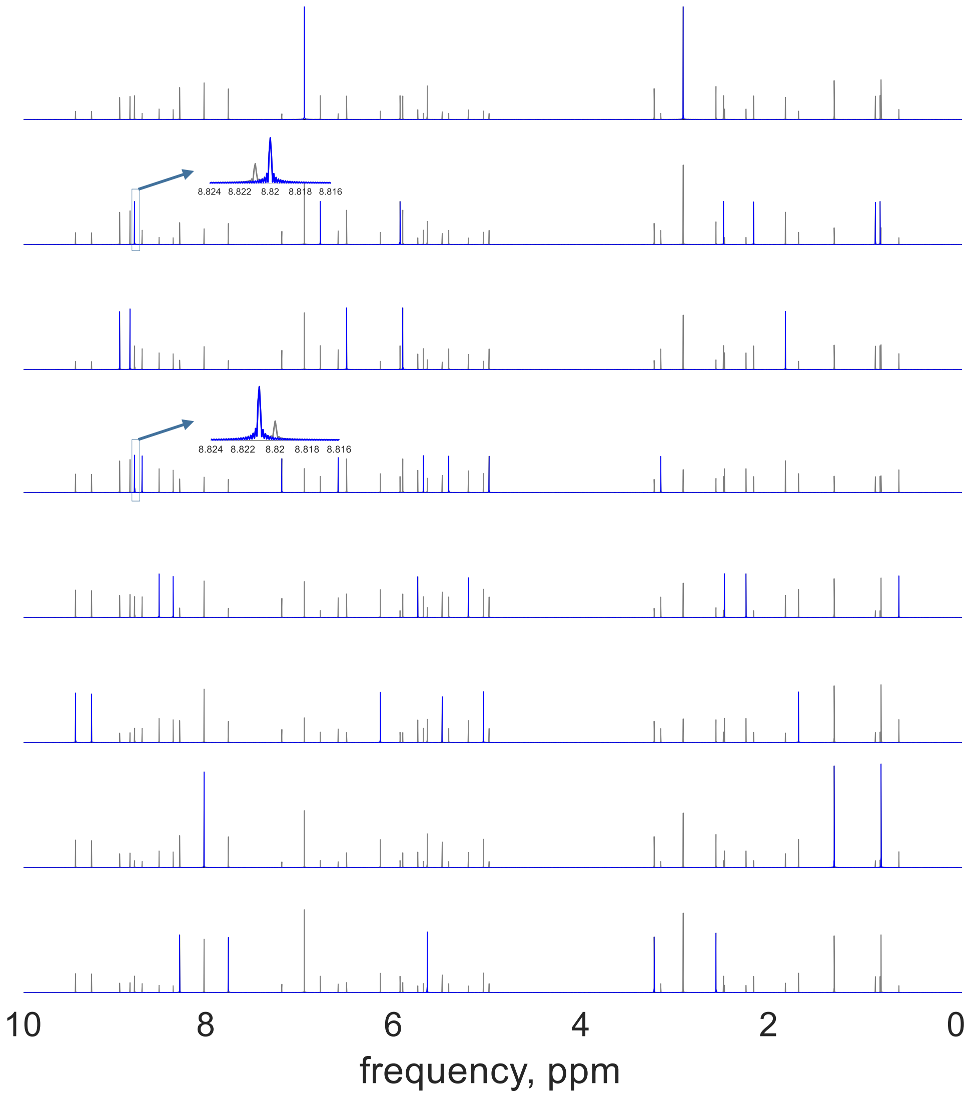

Figure S15 Signal decomposition of an 8-channel system using simulated FID assuming homogeneous B_0. Each channel is assigned a random number of spin, and random chemical shifts in the range $[0, 10]$ ppm (parts-per-million). The original signal is represented in grey, the split signal is represented in blue. Two peaks in channel-2 and channel-4 have only $\Delta f = 0.001$ ppm, see the zoomed-in view.

Supplementary Note 9. Wall clock time of the optimal control

To calculate the wall clock time of optimal control, the problem considers the pulse optimization for ^{1}H excitation, transfer I_z to -I_y. The pulse duration is 1 ms, and the pulse covers 20 kHz bandwidth. The nominal RF amplitude is 50 kHz with ±20% inhomogeneity, and the iteration stops when fidelity ≥ 0.995. The LBFGS-GRAPE algorithm was specified as the optimization method. Every time the optimization was started with a random guess for the shape phase, the shape amplitude was fixed. The hardware and software were detailed in the main text.

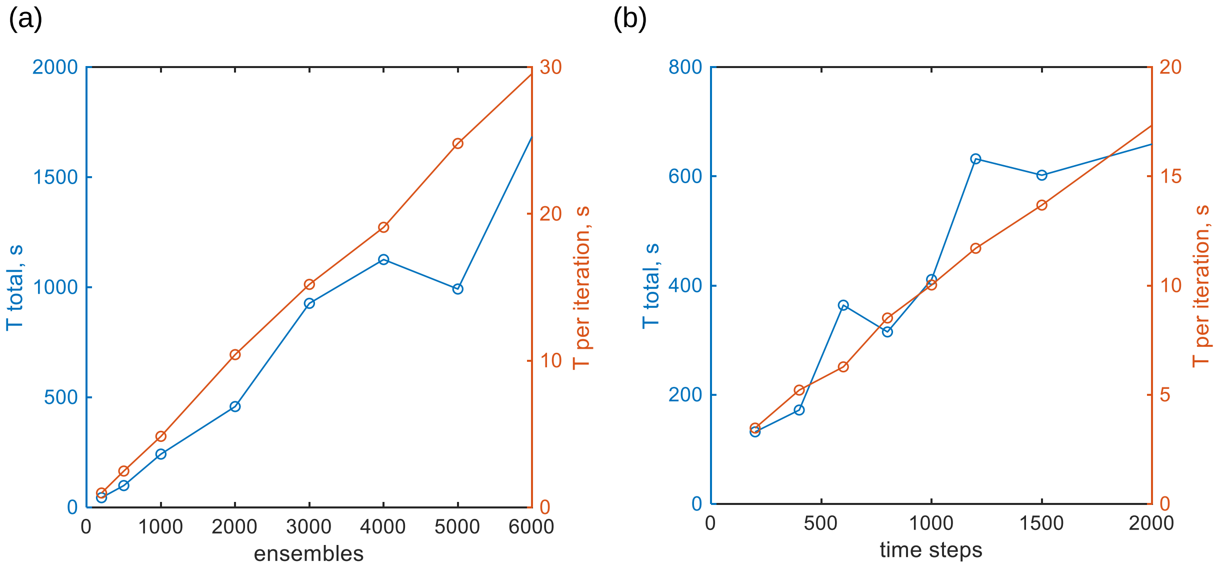

Figure S16 Wall clock time T of the optimal control algorithm in Spinach. (a) Time T used for an increasing number of ensembles, with 400 time steps. (b) Time T used for an increasing number of time steps, with 1000 ensembles.

Supplementary Note 10. Spin dynamics simulation for multiple samples

The spin dynamics calculation was completed in Spinach[7]. Generally, Spinach only supports defining one spin system or specifying one sample. To accommodate multiple samples, we suppose there is no coupling between different samples while neglecting the radiation damping, see supplementary S10. We define a composite spin system that includes all the isotopes and the corresponding interactions considered in each sample. An index was individually added to distinguish the isotopes from different samples.

To fix the variables in Spinach accounting for different samples and field inhomogeneity, we defined the variable arrays for the following physics:

- Spin state and spin operator

  The concerned spins of multiple samples were indexed, hence we could use the built-in functions to generate the physics for different samples.

  For example, `rho=state(spinSystem,'Lz',isoInd{m})` defines the m-th sample's spin state as I_z, where isoInd indicates the corresponding isotopes' index.

- Hamiltonian

  An internal Hamiltonian was generated for each voxel, including the local B_0 field, that is $H_0 = H_c + H_z \cdot B_{0i}$, where H_c is the spin coupling part, H_z is the Zeeman part calculated assuming a 1 T magnetic field, and B_{0i} is a local B_0 value. To accommodate the spins from multiple samples, the H_c and H_z were constructed by collecting all the sub-Hamiltonians into blocks of a block-diagonal matrix, and the corresponding spin state vectors were also stacked to match the Hamiltonian.

- Pulse sequences

  (1) Concerning the single pulse, the duration is 20 μs, and the excitation power was calibrated to achieve an average flip angle of 90°, assuming no RF coupling exists.

  (2) Concerning the optimal control pulse, it has a duration of 2 ms divided into 100 equal steps, and the excitation power is adjusted to examine the coupling effect.

  (3) For both the single pulse and optimal control pulse, the amplitude and phase of the pulse were computed based on the local B_1 field of each voxel, giving an overall excitation p at the TM network terminal.

Supplementary Note 11. Single channel optimal control

The pulse optimization for a single sample was completed in Spinach[7]. This simulation package contains an optimal control module based on the gradient ascent pulse engineering (GRAPE) method[8]. The B_0, B_1 field inhomogeneities, and resonance offset were considered in the optimal control.

To include the field inhomogeneity, the cylindrical sample was equally divided into multiple voxels along the sample axis (8 values in the z-direction), sample radius (3 values in the r-direction), and azimuthal (3 values in the θ-direction). The B_0 field was predicated using COMSOL's `mfnc` module accounting for the magnetic susceptibility mismatch under an 11.74 T background field, and then manually adjusted with a zero-order shim. The B_1 field was calculated from the EM simulation, supposing a reasonable power input. Each voxel was assigned a B_0 and a B_1 value, where B_0 was used to define the internal Hamiltonian, and B_1 was used to define the power level and phase of the pulse sequences.

When determining the optimal control parameters, the objective was to transfer each spin from I_z to I_x. Distinct parameters were fixed for the optimal pulse utilized in both simulation and experimental settings.

For simulations, expedited calculations were pursued. The resonance offset spanning $\nu_0 = \pm 2.5$ kHz, was subdivided into 20 segments. The resonance offsets were combined with the simulated field. The pulse sequence duration was set at 2 ms, segmented into 100 equivalent slices, with a fixed pulse amplitude of 1. The phase was varied, resulting in 100 variables.

For experiments, the optimal control pulses for ^{1}H excitation were examined. The resonance offset spanning $\nu_0 = \pm 15$ kHz, was divided into 100 segments. The nominal RF amplitude, $\nu_1 =$ 30kHz, exhibited $\pm 20\%$ inhomogeneities accounting for the simulated B_1 of a stripline, also divided into 100 segments. These resonance offsets were combined with B_1 inhomogeneities. The pulse amplitude was held constant at 1 while the phase was optimized. The pulse duration was 8 ms, divided into 4000 equivalent bins.

The control fidelity calculated the average value of the overlap between the final state and the target state in terms of all voxels, i.e., $\overline{\eta} = \overline{\langle \rho | I_x \rangle}$. The LBFGS-GRAPE algorithm was specified as the optimization method.

Here, we provide a spin dynamics description of the GRAPE algorithm[8] within the Liouvillian space, where the Hamiltonian is represented as the commutation superoperator and

the spin state is represented as a column vector. The Hamiltonian of the spin system can be written as the internal part plus the control part as

$$H(t) = H_{\text{int}} + H_{\text{rf}}(t) \tag{S26}$$

Where the internal part is the addition of the Zeeman part which is proportional to the local magnetic field and coupling part,

$$H_{\text{int}} = B_0 \cdot H_{\text{z0}} + H_c \tag{S27}$$

where H_{z0} is the time-independent Zeeman Hamiltonian within 1 T magnetic field, the B_0 indicates the local static field, H_c is set to zero when no spin coupling exists. The control part can be decomposed into x and y components, read as

$$H_{\text{rf}}(t) = \omega_x(t)I_x + \omega_y(t)I_y \tag{S28}$$

For the single-channel pulse, only one pair of operators is used, $\omega_x(t)$ and $\omega_y(t)$ are the control amplitude of RF pulse.

For numerical optimization, the period of the pulse is divided into N equal slices, i.e., $\tau = N\Delta t$, and the time-dependent Hamiltonian is approximated with the piecewise constant function. The spin dynamics can be described as the density matrix $\rho(t)$ evolves under the propagator $P(t)$. At the end of the pulse, the final spin state is given by[8]

$$\rho(\tau) = P_N \cdots P_2 P_1 \rho_0 \tag{S29}$$

where the k-step propagator P_k is

$$P_k = \exp[-i(H_{\text{int}} + H_{\text{rf,k}})\Delta t] \tag{S30}$$

where $H_{\text{rf},k} = \omega_{k,x}I_x + \omega_{k,y}I_y$. The fidelity function is measured by the inner product

$$\eta = Tr[I_{\text{x}}^{\dagger}\rho(\tau)] \tag{S31}$$

where I_{x} is the target state. The control variables were assigned with an initial guess and were updated according to the following formula

$$\omega^{s+1} = \omega^s - \alpha_s H_s^{-1}\nabla\eta \tag{S32}$$

Where $\nabla\eta$ is the gradient of the fidelity function, the gradient regards the k-step control is

$$\frac{\partial\eta}{\partial\omega_k} =< I_{\text{x}}|P_N \cdots P_{k+1}\frac{\partial P_k}{\partial\omega_k}P_{k-1} \cdots P_1|\rho_0 > \tag{S33}$$

The Spinach uses auxiliary matrix formalism[9] to calculate Eq. S33. The H_s^{-1} is the approximate inverse Hessian matrix, calculated from a stack of history controls and gradients, the so-called LBFGS algorithm[10]. The α_s is calculated from a line search which finds an optimization step in the specified direction[11]. The variables updating stops when the iteration termination condition is satisfied, i.e. $\eta > 0.995$.

To generate a pulse robust to the resonance offsets and RF inhomogeneity, the ensemble fidelity was considered,

$$\overline{\eta} = \frac{1}{n_{\text{rf}} n_{\text{off}}} \sum_{n_{\text{rf}}} \sum_{n_{\text{off}}} \eta. \tag{S34}$$

Herem n_{rf}, n_{off}, represents the number of RF amplitudes and resonance offsets, respectively. Similarly, for using Eq. S32, the overall gradient is given by taking the mean value of each ensemble gradient $\nabla\eta$.

Supplementary Note 12. Variables index

variable	description
a	incident wave
b	reflected wave
B_0	static magnetic field
B_1	radio frequency magnetic field
C_A	figure of merit of signal decomposition
E	identity matrix
f_{ij}	FID from sample j to coil i
$\hat{F}$	coupling matrix estimated from BSS method
F	matrix transform excitation wave to coil current
G	signal gain matrix in the reception stage
I	current on the coil
p_0	optimal control pulse
p	cooperative pulse
s	source signals in the BSS model
S_C	S matrix of coil array
S_M	S matrix of matching network
S_{CM}	S matrix of combined coil and matching network
$\hat{V}_n$	noise covariance
$\hat{V}_s$	signal covariance
x	detected signals in the BSS model
δ	compensation pulse term
Δf	spectral difference
η	transfer fidelity of the pulse
ν_0	resonance offset
ν_1	nutation frequency or RF amplitude in Hz

Supplementary Table 3 Variables used in the article.

Supplementary Note 13. Parallel NMR experiment setup

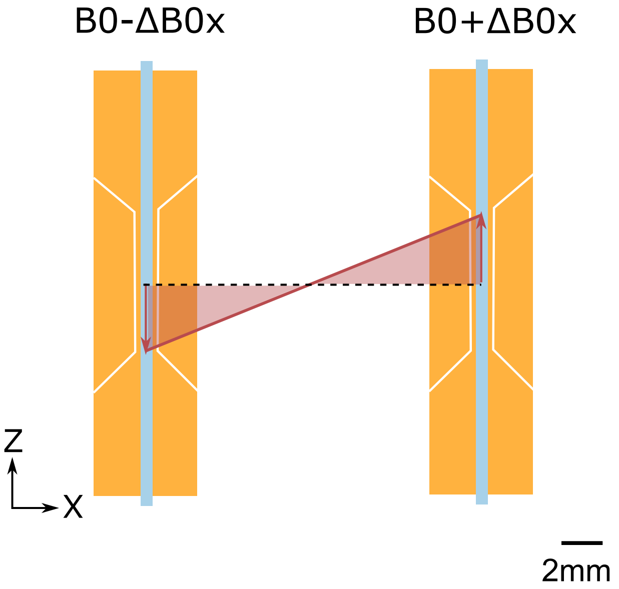

Figure S17 Set-up for a parallel signal acquisition experiment. Two striplines were inserted into the magnet system, with a global shim set to 1000 Hz/cm along the x-axis, to separate the two water peaks. As a result, the static magnetic field in the two samples were $B_0 \pm \Delta B_{0x}$.

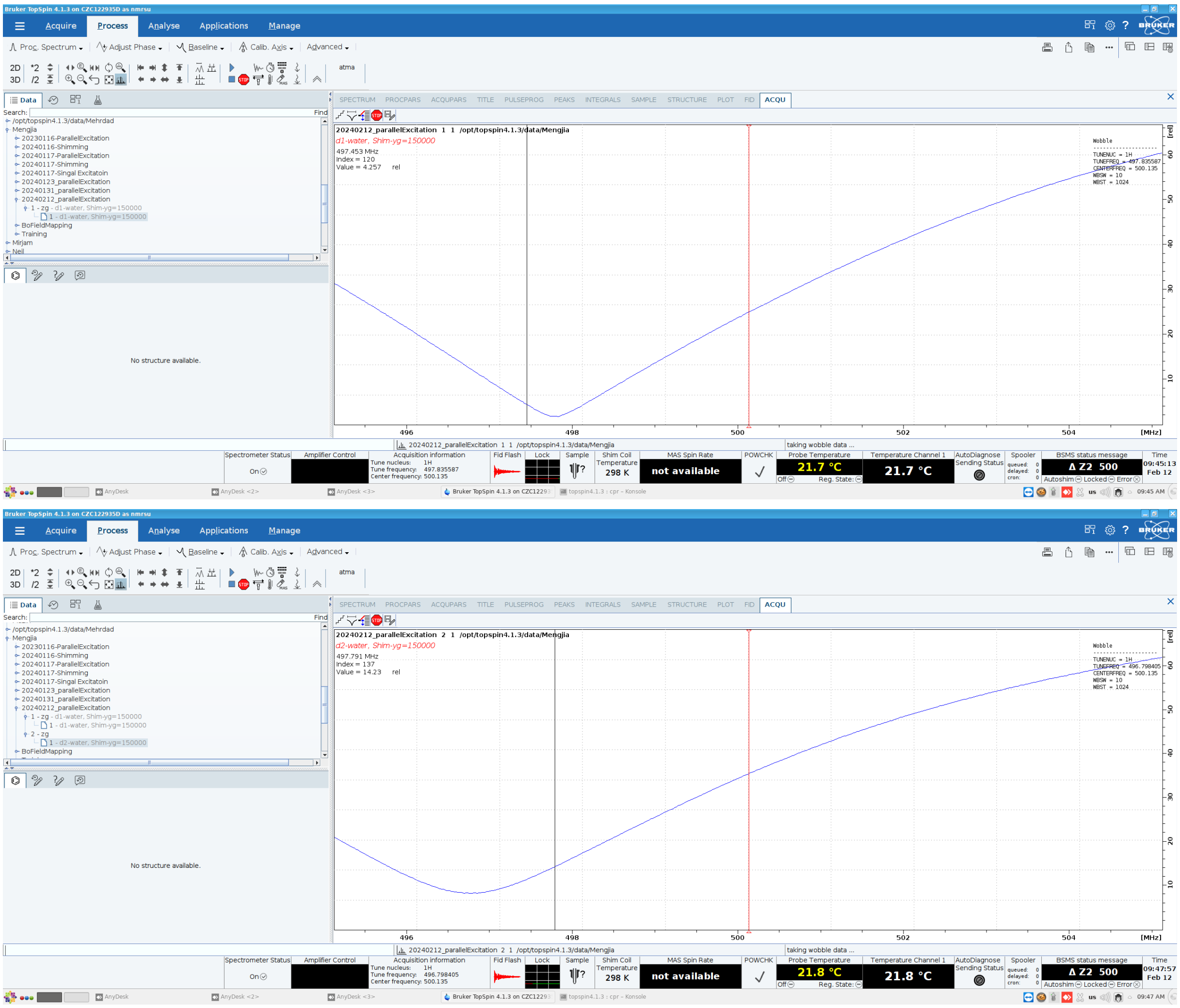

Figure S18 Tuning and matching of two detectors for the parallel pulse compensation experiments.

Supplementary Note 14. Chemical shifts of samples

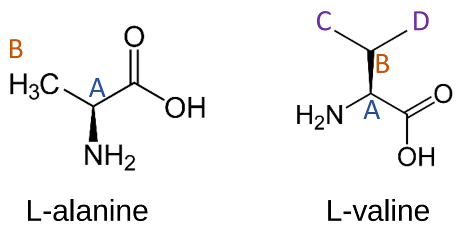

Figure S19 Chemical structure of L-alanine (CAS No. 56-41-7) and L-valine (CAS No. 516-06-3).

Symbol	L-alanine	L-valine
A	3.787	3.608
B	1.482	2.260
C		1.045
D		0.995

Supplementary Table 4 Chemical shifts in parts-per-million of L-alanine and L-valine.

Supplementary References

1. Cheng, Y.-T., Jouda, M. & Korvink, J. Sample-centred shimming enables independent parallel NMR detection. *Scientific Reports* **12**, 14149 (2022).

2. Lagarias, J. C., Reeds, J. A., Wright, M. H. & Wright, P. E. Convergence properties of the nelder–mead simplex method in low dimensions. *SIAM Journal on Optimization* **9**, 112–147 (1998).

3. Warnick, K. & Jensen, M. Effects of mutual coupling on interference mitigation with a focal plane array. *IEEE Transactions on Antennas and Propagation* **53**, 2490–2498 (2005).

4. Tošner, Z. *et al.* Overcoming volume selectivity of dipolar recoupling in biological solid-state NMR spectroscopy. *Angewandte Chemie International Edition* **57**, 14514–14518 (2018).

5. Mao, X.-A. & Ye, C.-H. Understanding radiation damping in a simple way. *Concepts in Magnetic Resonance* **9**, 173–187 (1997).

6. Belouchrani, A., Abed-Meraim, K., Cardoso, J.-F. & Moulines, E. A blind source separation technique using second-order statistics. *IEEE Transactions on Signal Processing* **45**, 434–444 (1997).

7. Hogben, H. J., Krzystyniak, M., Charnock, G. T., Hore, P. J. & Kuprov, I. Spinach–a software library for simulation of spin dynamics in large spin systems. *Journal of Magnetic Resonance* **208**, 179–194 (2011).

8. Khaneja, N., Reiss, T., Kehlet, C., Schulte-Herbruggen, T. & Glaser, S. J. Optimal control of coupled spin dynamics: Design of NMR pulse sequences by gradient ascent algorithms. *Journal of Magnetic Resonance* **172**, 296–305 (2005).

9. Goodwin, D. L. & Kuprov, I. Auxiliary matrix formalism for interaction representation transformations, optimal control, and spin relaxation theories. *The Journal of Chemical Physics* **143**, 084113 (2015).

10. Byrd, R. H., Nocedal, J. & Schnabel, R. B. Representations of quasi-Newton matrices and their use in limited memory methods. *Mathematical Programming* **63**, 129–156 (1994).

11. FMINLBFGS: Fast Limited Memory Optimizer. https://ww2.mathworks.cn/matlabcentral/fileexchange/23245-fminlbfgs-fast-limited-memory-optimizer (2024).
